# Optimizing Multivitamin Supplementation for Sleeve Gastrectomy Patients

**DOI:** 10.1007/s11695-021-05282-4

**Published:** 2021-02-23

**Authors:** Laura Heusschen, Agnes A. M. Berendsen, Mellody I. Cooiman, Laura N. Deden, Eric J. Hazebroek, Edo O. Aarts

**Affiliations:** 1 Department of Bariatric Surgery, Vitalys, part of Rijnstate Hospital, PO box 9555, 6800 Arnhem, The Netherlands; 2 Divison of Human Nutrition and Health, Wageningen University, Wageningen, The Netherlands

**Keywords:** Morbid obesity, Bariatric surgery, Metabolic surgery, Sleeve gastrectomy (SG), Deficiencies, Micronutrients, Vitamins, Minerals, Supplementation

## Abstract

**Purpose:**

Micronutrient deficiencies are frequently reported after sleeve gastrectomy (SG), and therefore lifelong daily multivitamin supplementation is highly recommended. Based on literature and the results of a previous randomized controlled trial, a specialized multivitamin supplement for SG patients was further optimized (WLS Optimum 2.0, FitForMe). The present study reports on its short-term effectiveness.

**Materials and Methods:**

An open-label study was performed in which 76 patients were included to receive WLS Optimum 2.0 for 12 months (Opt 2.0 group). This group was compared with a group of 75 patients that had received WLS Optimum 1.0 for 12 months during a previous study (Opt 1.0 group).

**Results:**

Intention-to-treat analysis (Opt 1.0, *n* = 69; Opt 2.0, *n* = 75) showed higher serum levels of vitamin B12, vitamin B6, and zinc, and a lower prevalence of deficiencies for vitamin B12 and phosphate in the Opt 2.0 group. MCV and serum folic acid levels were higher in the Opt 1.0 group. Over the 12-month study period, mean increase in serum levels of phosphate, vitamin B6, and zinc was higher in the Opt 2.0 group, and MCV and serum vitamin D levels increased more in the Opt 1.0 group.

**Conclusion:**

The present study showed that the use of a specialized multivitamin supplement for SG patients is effective at preventing deficiencies for most vitamins and minerals, specifically in compliant patients. However, a strict follow-up regime remains necessary to monitor nutritional status and to improve patient compliance.

**Supplementary Information:**

The online version contains supplementary material available at 10.1007/s11695-021-05282-4.

## Introduction

The laparoscopic sleeve gastrectomy (SG) is currently the most commonly performed bariatric procedure worldwide [1]. Whereas the impact of more malabsorptive procedures such as the Roux-en-Y gastric bypass (RYGB) on nutritional status is well known, the occurrence of nutritional deficiencies after SG is often underestimated [2–4]. After SG, there are several factors that put patients at risk for developing nutritional deficiencies, including reduced dietary intake, decreased hydrochloric acid and intrinsic factor secretion, poor food choices and food intolerance [5, 6]. Although prevalence estimates vary widely, micronutrient deficiencies including vitamin D (5-89%), vitamin B12 (9-26%) and iron (12-43%), and elevated parathyroidhormone levels (PTH; 14-39%) have been frequently reported in the first year post-SG [7–11]. To prevent patients from developing these micronutrient deficiencies, lifelong daily multivitamin supplementation is highly recommended [12].

In 2012, a customized multivitamin supplement with elevated doses of vitamins and minerals specifically designed for SG patients was introduced (WLS Optimum 1.0; FitForMe, Rotterdam, the Netherlands). In a double-blind randomized controlled trial (RCT) [13], WLS Optimum 1.0 has shown to be effective in reducing the prevalence of anemia and improving serum levels of folic acid, PTH, and vitamin B1 in comparison to a standard, over-the-counter multivitamin supplement (100% RDA) [13]. No differences were found for the prevalence of deficiencies for iron, vitamin B12, vitamin D, and other vitamins and minerals. Based on these findings, the composition of WLS Optimum 1.0 was further optimized by elevating the levels of elementary iron, folic acid, vitamin B12, vitamin B1, copper, and zinc. The present study reports on the short-term effectiveness (≤ 12 months) of WLS Optimum 2.0 in comparison to WLS Optimum 1.0.

## Methods

### Study design

The present study combines data of two prospective studies, i.e. the VITAAL I study and the VITAAL II study. The VITAAL I study was a double-blind RCT, in which included patients received either WLS Optimum 1.0 (intervention group) or a standard, over-the-counter multivitamin supplement (sMVS, control group) for 12 months [13]. All 75 patients who received WLS Optimum 1.0 were included in this study (Opt 1.0 group). During the VITAAL II study, 76 new patients were included to receive WLS Optimum 2.0 for 12 months (Opt 2.0 group). Exclusion criteria were creatinine > 150 μmol/L, systemic diseases that affect the gastrointestinal tract, psychiatric illness, the use of drugs that affect bone metabolism, and known pregnancy during the study period. All SG procedures were performed by experienced bariatric surgeons, using the standardized operating technique as described earlier [13].

Both study protocols were approved by the Medical Ethics Review Committee of the Radboud University Medical Centre and the Local Ethical Committee of Rijnstate Hospital Arnhem and were conducted in concordance with the principles of the Declaration of Helsinki. The initial RCT was registered at the clinical trials registry of the National Institutes of Health (ClinicalTrials.gov; identifier NCT01609387).

### WLS Optimum

WLS Optimum is a customized MVS for SG patients and contains elevated doses of multiple vitamins and minerals. The contents of WLS Optimum 1.0 and 2.0 are shown in Table 1. Both supplements were similar in color and size. They had the exact same raw base compounds and cherry-flavored capsule. In comparison to its previous version, the Optimum 2.0 supplement contained higher levels of elementary iron, folic acid, vitamin B12, vitamin B1, copper, and zinc and a lower level of vitamin A. For both supplements, patients were instructed to take one capsule per day, starting from the day of surgery. Instructions on intake were given before surgery and at all medical checkups postoperatively. In addition, patients were instructed to take calcium/cholecalciferol 500/800 two times a day as part of the standard post-SG treatment protocol.

**Table 1:** Content of WLS Optimum 1.0 and WLS Optimum 2.0

	Optimum 1.0		Optimum 2.0	
Micronutrients	Dose	RDA (%)	Dose	RDA (%)
*Vitamins*				
Vitamin A, mg	1.00	125.0	0.80	100.0
Vitamin B1, mg	2.00	182.0	2.75	250.0
Vitamin B2, mg	2.00	143.0	2.00	143.0
Vitamin B3, mg	25.00	156.0	25.00	156.0
Vitamin B5, mg	9.00	150.0	9.00	150.0
Vitamin B6, mg	2.00	143.0	2.00	143.0
Biotin, μg	150.00	300.0	150.00	300.0
Folic acid, μg	300.00	150.0	500.00	250.0
Vitamin B12, μg	10.00	400.0	100.00	4000.0
Vitamin C, mg	100.00	125.0	100.00	125.0
Vitamin D, μg	7.50	150.0	7.50	150.0
Vitamin E, mg	12.00	100.0	12.00	100.0
Vitamin K1, μg	90.00	120.0		
*Minerals*				
Chrome, μg	40.00	100.0	40.00	100.0
Iron, mg	21.00	150.0	28.00	200.0
Iodine, μg	150.00	100.0	150.00	100.0
Copper, mg	1.00	100.0	1.90	190.0
Magnesium, mg	30.00	8.0		
Manganese, mg	3.00	150.0	3.00	150.0
Molybdenum, μg	50.00	100.0	50.00	100.0
Selenium, μg	55.00	100.0	55.00	100.0
Zinc, mg	15.00	150.0	28.00	280.0

*RDA* recommended daily allowance.

### Data Collection

All patients visited the hospital for standard laboratory blood tests and anthropometric measurements during regular visits before surgery (T0) and at 6 months (T6) and 12 months (T12) after surgery. Blood was collected by venipuncture. The following blood parameters were measured on random access analyzers: hemoglobin, mean corpuscular volume (MCV; XN-10 Sysmex); ferritin, folic acid, vitamin B12, 25-OH vitamin D and PTH (Modular E170, Roche); and phosphate, calcium, magnesium, and albumin (Modular P800, Roche). Zinc was analyzed by inductively coupled plasma mass spectrometry (Shimadzu). Vitamin B1 and vitamin B6 were analyzed on a high-performance liquid chromatography with fluorescence detector (Shimadzu). Calcium levels were corrected for albumin using the following equation: Ca_corr_ = total calcium – (0.025 x albumin) + 1.

A deficiency was defined as a serum level below the local reference value (reference values in tables). Serum ferritin levels were used for the diagnosis of iron deficiency. Preoperative vitamin B12 and vitamin D deficiencies were treated with predefined medication. In case of a nutritional deficiency after surgery, treatment was performed according to local protocol as described earlier [14]. Subsequent data of the corresponding parameter were excluded.

Weight loss was expressed as excess body weight loss (EWL) and total body weight loss (TWL). EWL was calculated as [weight loss/excess weight based on ideal body weight at BMI 25 kg/m^2^ × 100%]. TWL was calculated as [weight loss/initial weight × 100%].

### Statistical Analyses

General characteristics of the Opt 1.0 and Opt 2.0 group were compared using independent sample *t* tests for continuous data and Chi-Square tests for discrete data (or Fisher’s Exact test when more than 20% of expected counts were below 5).

Serum levels of ferritin, folic acid, PTH, vitamin B1, and vitamin B6 were transformed to natural logarithms before analysis. Differences in mean serum levels at T6 and T12 were tested using one-way analysis of covariance (ANCOVA) with baseline serum level as covariate. Analyses of vitamin B12 and vitamin D were not corrected by baseline serum level as baseline deficiencies were treated before surgery. The number of deficiencies between the groups at T6 and T12 were compared using Chi-Square tests (or Fisher’s Exact test when more than 20% of expected counts were below 5). Linear mixed models were used to assess if serum levels changed differently over time between the groups. Follow-up measurements of patients who became pregnant or underwent revisional surgery were excluded from the analyses.

Intention-to-treat (ITT) analysis was performed as the primary analysis. Additionally, a per-protocol (PP) analysis was performed, excluding all patient who reported to not use the assigned supplement.

All statistical analyses were performed using IBM SPSS Statistics 25 for Windows (IBM Corp., Armonk USA).

## Results

The total study population consisted of 151 patients. Seven patients were excluded because they did not complete any of the follow-up measurements during the 12-month study period. In total, 144 patients were available for the ITT analysis: 69 patients receiving Optimum 1.0 (Opt 1.0 group) and 75 patients receiving Optimum 2.0 (Opt 2.0 group). For the PP analysis, 44 patients (63.8%) reported to use Optimum 1.0 and 50 patients reported to use Optimum 2.0 (66.7%) at T6. At T12, this number decreased to 38 (55.1%) and 41 patients (54.7%), respectively.

General characteristics of patients in the Opt 1.0 and Opt 2.0 group are shown in Table 2. Both groups were similar with respect to age (38.2 ± 12.4 years vs. 38.1 ± 12.9 years), gender (73.9% vs. 77.3% female), preoperative body weight (141.3 ± 26.1 kg vs. 140.5 ± 28.2 kg) and BMI (47.6 ± 9.0 kg/m^2^ vs. 47.1 ± 7.9 kg/m^2)^, and comorbidities (*P* > 0.05 for all). In nine patients, a gastric band was removed before conversion to SG (4.3 vs. 8.0%).

**Table 2:** General characteristics of the Opt 1.0 group and Opt 2.0 group

	Optimum 1.0 (*n* = 69)		Optimum 2 .0 (*n* = 75)	
Age (years)	38.2	± 12.4	38.1	± 12.9
Gender (female)	51	(73.9)	58	(77.3)
Body weight before surgery (kg)	141.3	± 26.1	140.5	± 28.2
BMI before surgery (kg/m^2^)	47.6	± 9.0	47.1	± 7.9
Previous LAGB	3	(4.3)	6	(8.0)
Comorbidities				
*T2DM*	9	(13.0)	9	(12.0)
*Hypertension*	15	(21.7)	22	(29.3)
*Dyslipidemia*	3	(4.3)	8	(10.7)
*OSAS*	7	(10.1)	8	(10.7)
BMI at 12 months after surgery (kg/m^2^)	32.7	± 7.2	31.9	± 6.3
EWL at 12 months after surgery (%)	70.5	± 22.7	72.2	± 20.6
TWL at 12 months after surgery (%)	31.3	± 8.6	32.1	± 8.7

Data are presented as mean ± standard deviation and frequencies (percentages).

*BMI*, Body Mass Index; *LAGB,* Laparoscopic adjustable gastric banding; *T2DM,* Type 2 Diabetes Mellitus; *OSAS,* Obstructive Sleep Apnea Syndrome; *EWL,* Excess Weight Loss; *TWL*, Total Weight Loss.

*P *> 0.05 for all outcomes.

The degree of body weight loss after 12 months was similar for patients in the Opt 1.0 group and the Opt 2.0 group with a mean BMI of 32.7 ± 7.2 kg/m^2^ vs. 31.9 ± 6.3 kg/m^2^, EWL of 70.5 ± 22.7% vs. 72.2 ± 20.6% and TWL of 31.3 ± 8.6% vs. 32.1 ±8.7%, respectively (*P* > 0.05 for all).

The use of medication known to cause drug-nutrient interactions at T12 (e.g., proton pump inhibitors, metformin) was also comparable between the groups (28.8 vs. 34.4%).

### Hemoglobin Metabolism

Mean serum levels and prevalence of deficiencies regarding hemoglobin, MCV, ferritin, folic acid, and vitamin B12 can be found in Table 3**.** At baseline, mean serum levels and prevalence of pre-operative deficiencies were similar between the groups.

**Table 3:** Mean serum levels and prevalence of deficiencies of Hemoglobin, MCV, Ferritin, Folic acid, and Vitamin B12.

Serum variables (reference values)	Type MVS	Serum levels				Deficiencies		
		T0	T6	T12	Δ (T12-T0)	T0	T6	T12
**Hemoglobin** (M: 8.4–10.8 mmol/L F: 7.4–9.9 mmol/L)	**Optimum 1.0** (*n* = 69/67/66)	8.7 ± 0.8	8.6 ± 0.8	8.4 ± 0.7	-0.2 ± 0.6	4 (5.8)	3 (4.5)	7 (10.6)
	**Optimum 2.0** (*n* = 75/70/64)	8.8 ± 0.8	8.5 ± 0.7	8.4 ± 0.8	-0.3 ± 0.6	3 (4.0)	6 (8.6)	6 (9.4)
**MCV** (80–100 fL)	**Optimum 1.0** (*n* = 68/67/66)	88.7 ± 4.1	90.5 ± 3.3	91.8 ± 4.4	+3.2 ± 4.2	1 (1.5)	0 (0.0)	0 (0.0)
	**Optimum 2.0** (*n* = 75/68/64)	88.8 ± 3.8	89.7 ± 3.6	89.8 ± 4.0^***^	+0.9 ± 3.4^**^	1 (1.3)	0 (0.0)	1 (1.6)
**Ferritin** (20–300 ng/mL)	**Optimum 1.0** (*n* = 69/53/65)	127.6 ± 96.4	149.0 ± 114.0	139.4 ± 104.7	+8.1 ± 55.4	2 (2.9)	0 (0.0)	2 (3.1)
	**Optimum 2.0** (*n* =75/67/63)	119.8 ± 99.3	146.8 ± 109.7	124.1 ± 101.8	+3.5 ± 54.0	1 (1.3)	1 (1.5)	7 (11.1)
**Folic acid** (9.1–36 nmol/L)^1^	**Optimum 1.0** (*n* =68/66/66)	16.6 ± 6.7	22.3 ± 9.5	21.8 ± 10.0	+5.1 ± 9.2	2 (2.9)	5 (7.6)	5 (7.6)
	**Optimum 2.0** (*n* =75/68/63)	14.9 ± 6.2	18.3 ± 10.1^*^	19.7 ± 13.4	+4.8 ± 13.5	0 (0.0)	3 (4.4)	5 (7.9)
**Vitamin B12** (200–570 pmol/L)^2^	**Optimum 1.0** (*n* =67/52/59)	289.8 ± 96.4	276.1 ± 84.6	267.3 ± 80.0	-32.9 ± 76.2	1 (1.5)	11 (21.2)	15 (25.4)
	**Optimum 2.0** (*n* =71/65/57)	299.7 ± 95.6	310.8 ± 94.6^*^	302.4 ± 93.2^*^	+5.5 ± 103.7	1 (1.4)	7 (10.8)	6 (10.5)^*^

Data are presented as mean ± standard deviation and frequencies (percentages). *MVS,* multivitamin supplement; *MCV,* mean corpuscular volume.

^1^Reference range for the assay in the VITAAL II study (Optimum 2.0) was 6-28 nmol/L

^2^Reference range before surgery (T0) was 145 – 570 pmol/L.

^*^P<0.05, ^**^P<0.01, ^***^P<0.001 for Optimum 1.0 vs. Optimum 2.0

MCV increased over time in the Opt 1.0 group, but not in the Opt 2.0 group (+3.2 ± 4.2 fL vs. +0.9 ± 3.4 fL, *P* = 0.002), resulting in a significantly lower MCV in the Opt 2.0 group at T12 (89.8 ± 4.0 fL vs. 91.8 ± 4.4 fL, *P* < 0.001).

At T6, mean serum folic acid level was lower in the Opt 2.0 group than in the Opt 1.0 group (18.3 ± 10.1 nmol/L vs. 22.3 ± 9.5 nmol/L, *P* = 0.047). This difference was no longer present at T12 (19.7 ± 13.4 nmol/L vs. 21.8 ± 10.0 nmol/L, *P* = 0.15). The prevalence of deficiencies did not differ between the groups during the study period.

Mean serum vitamin B12 levels were higher in the Opt 2.0 group compared with the Opt 1.0 group at both T6 (310.8 ± 94.6 pmol/L vs. 276.1 ± 84.6 pmol/L, *P* = 0.041) and T12 (302.4 ± 93.2 pmol/L vs. 267.3 ± 80.0 pmol/L, *P* = 0.031). At T12, the prevalence of vitamin B12 deficiencies was also lower in the Opt 2.0 group (10.5% vs. 25.4%, *P* = 0.037). Over time, serum vitamin B12 levels increased in the Opt 2.0 group while they decreased in the Opt 1.0, group but this difference was not statistically significant (+5.5 ± 103.7 pmol/L vs. −32.9 ± 76.2 pmol/L, *P *= 0.18).

No significant differences were observed between the groups for hemoglobin and ferritin. In the PP analysis, only the observed differences for MCV and serum vitamin B12 level at T12 were statistically significant (Supplementary Table 1).

### Calcium and Vitamin D Metabolism

Mean serum levels and prevalence of deficiencies regarding vitamin D, PTH, calcium, magnesium, phosphate and albumin can be found in Table 4**.**

**Table 4:** Mean serum levels and prevalence of deficiencies of Vitamin D, PTH, Calcium, Magnesium, Phosphate and Albumin

Serum variables (reference values)	Type MVS	Serum levels				Deficiencies		
		T0	T6	T12	Δ (T12-T0)	T0	T6	T12
**Vitamin D** (> 50 nmol/L)	**Optimum 1.0** (*n*=69/66/64)	36.6 ± 21.8	86.7 ± 27.6	84.5 ± 32.3	+48.8 ± 29.0	51 (73.9)	5 (7.6)	7 (10.9)
	**Optimum 2.0** (*n*=75/68/63)	55.8 ± 24.7^***^	92.5 ± 24.1	86.2 ± 22.5	+28.6 ± 23.4^***^	29 (38.7)^***^	1 (1.5)	3 (4.8)
**PTH** (1.3–6.8 pmol/L)	**Optimum 1.0** (*n*=69/66/65)	3.7 ± 2.0	3.4 ± 1.6	3.5 ± 1.9	-0.3 ± 2.1	7 (10.1)^1^	1 (1.5)^1^	4 (6.2)^1^
	**Optimum 2.0** (*n*=75/68/61)	3.3 ± 2.0	3.1 ± 1.7	3.5 ± 2.0	+0.4 ± 1.6	2 (2.7)^1^	1 (1.5)^1^	4 (6.6)^1^
**Calcium**^2^ (2.10–2.55 mmol/L)^3^	**Optimum 1.0** (*n*=62/65/65)	2.35 ± 0.11	2.40 ± 0.08	2.40 ± 0.08	+0.05 ± 0.11	1 (1.6)	0 (0.0)	0 (0.0)
	**Optimum 2.0** (*n*=66/70/61)	2.34 ± 0.10	2.42 ± 0.08	2.39 ± 0.09	+0.05 ± 0.09	6 (9.1)	0 (0.0)	1 (1.6)
**Magnesium** (0.70-1.10 mmol/L)	**Optimum 1.0** (*n*=63/40/43)	0.80 ± 0.07	0.82 ± 0.05	0.83 ± 0.06	+0.03 ± 0.07	3 (4.8)	1 (2.5)	1 (2.3)
	**Optimum 2.0** (*n*=64/37/20)	0.80 ± 0.06	0.82 ± 0.05	0.81 ± 0.06	+0.01 ± 0.06	4 (6.3)	1 (2.7)	1 (5.0)
**Phosphate** (0.87-–1.45 mmol/L)^4^	**Optimum 1.0** (*n*=62/49/55)	0.95 ± 0.18	1.01 ± 0.15	1.02 ± 0.20	+0.07 ± 0.20	22 (35.5)	10 (20.4)	10 (18.2)
	**Optimum 2.0** (*n*=63/42/27)	0.85 ± 0.16^**^	1.02 ± 0.15	1.05 ± 0.20	+0.17 ± 0.25^**^	17 (27.0)	2 (4.8)^*^	1 (3.7)
**Albumin** **(**35–50 g/L)	**Optimum 1.0** (*n*=63/65/65)	37.8 ± 3.8	38.8 ± 3.4	38.5 ± 3.2	+1.0 ± 3.5	9 (14.3)	6 (9.2)	8 (12.3)
	**Optimum 2.0** (*n*=66/70/62)	38.2 ± 2.9	38.2 ± 2.9	38.2 ± 2.9	-0.02 ± 2.6	5 (7.6)	5 (7.1)	6 (9.7)

Data are presented as mean ± standard deviation and frequencies (percentages). *MVS,* multivitamin supplement*; PTH,* parathyroidhormone

^1^Elevated PTH levels

^2^Corrected for albumin levels (total calcium – (0.025 x albumin) + 1).

^3^Reference range for the assay in the VITAAL II study (Optimum 2.0) was 2.20-2.65 mmol/L

^4^Reference range for the assay in the VITAAL II study (Optimum 2.0) was 0.80-1.40 mmol/L

^*^P<0.05, ^**^P<0.01, ^***^P<0.001 for Optimum 1.0 vs. Optimum 2.0.

At baseline, mean serum vitamin D level was higher in the Opt 2.0 group than in the Opt 1.0 group (55.8 ± 24.7 nmol/L vs. 36.6 ± 21.8 nmol/L, *P* < 0.001), and the prevalence of vitamin D deficiencies was also lower in this group (respectively 38.7% vs. 73.9%, *P* < 0.001). Over time, mean increase in serum vitamin D level was lower in Opt 2.0 group than in the Opt 1.0 group (+28.6 ± 23.4 nmol/L vs. +48.8 ± 29.0 nmol/L, *P* < 0.001), and the differences in vitamin D serum levels and deficiencies were no longer present at T6 and T12.

Although mean serum phosphate level at baseline was lower in the Opt 2.0 group compared with the Opt 1.0 group (0.85 ± 0.16 mmol/L vs. 0.95 ± 0.18 mmol/L, *P = *0.002), the prevalence of phosphate deficiencies at T6 was lower in the Opt 2.0 group (4.8% vs. 20.4%, *P *= 0.028). At T12, this prevalence was 3.7% vs. 18.2% (*P *= 0.09). Over time, mean increase in phosphate level was higher in the Opt 2.0 group than in the Opt 1.0 group (+0.17 ±0.25 mmol/L vs. +0.07 ±0.20 mmol/L, *P *= 0.003).

The ITT analysis demonstrated no differences for PTH, calcium, magnesium, and albumin between both groups. In contrast, the PP analysis showed significantly different changes in serum levels of PTH, calcium, and albumin between the two groups over the study period (Supplementary Table 2). Mean increase in serum level was higher in the Opt 2.0 group than in the Opt 1.0 group for PTH (+0.5 ± 1.4 pmol/L vs. −0.3 ± 1.9 pmol/L, *P* = 0.014) and calcium (+0.06 ± 0.09 mmol/L vs. + 0.05 ± 0.11 mmol/L, *P* = 0.037) but lower for albumin (−0.03 ± 2.6 g/L vs. +1.3 ± 3.0 g/L, *P* = 0.016).

### Vitamin B1, Vitamin B6, and Zinc

Mean serum levels and prevalence of deficiencies regarding vitamin B1, vitamin B6, and zinc can be found in Table 5**.**

**Table 5:** Mean serum levels and prevalence of deficiencies of Vitamin B1 and B6, and Zinc

Serum variables (reference values)	Type MVS	Serum levels				Deficiencies		
		T0	T6	T12	Δ (T12-T0)	T0	T6	T12
**Vitamin B1** (95–175 nmol/L)	**Optimum 1.0** (*n*=61/49/54)	167.8 ± 29.5	148.0 ± 27.6	145.4 ± 29.8	-19.3 ± 40.6	0 (0.0)	1 (2.0)	2 (3.7)
	**Optimum 2.0** (*n*=70/39/22)	171.4 ± 28.2	144.8 ± 29.2	153.9 ± 36.5	-14.6 ± 37.5	0 (0.0)	1 (2.6)	0 (0.0)
**Vitamin B6** (25–100 nmol/L)	**Optimum 1.0** (*n*=61/49/54)	79.3 ± 24.0	91.7 ± 36.1	82.9 ± 27.3	+3.1 ± 26.6	0 (0.0)	0 (0.0)	0 (0.0)
	**Optimum 2.0** (*n*=70/39/21)	75.0 ± 23.0	87.1 ± 31.3	99.8 ± 31.7^*^	+25.7 ± 29.7^**^	0 (0.0)	0 (0.0)	0 (0.0)
**Zinc** (9.2–18.4 μmol/L)	**Optimum 1.0** (*n*=61/48/53)	12.2 ± 1.6	11.7 ± 1.6	11.7 ± 1.9	-0.4 ± 2.2	1 (1.6)	3 (6.3)	2 (3.8)
	**Optimum 2.0** (*n*=70/38/21)	11.2 ± 2.3^**^	12.9 ± 2.2^**^	12.6 ± 2.1	+1.3 ± 3.9^***^	12 (17.1)^**^	2 (5.3)	1 (4.8)

Data are presented as mean ± standard deviation and frequencies (percentages). *MVS,* multivitamin supplement.

^*^P<0.05, ^**^P<0.01, ^***^P<0.001 for Optimum 1.0 vs. Optimum 2.0.

Mean change in serum vitamin B6 level was greater in the Opt 2.0 group than in the Opt 1.0 group (+25.7 ± 29.7 nmol/L vs. +3.1 ± 26.6 nmol/L, *P* = 0.008), resulting in a significantly higher mean serum vitamin B6 level in this group at T12 (99.8 ± 31.7 nmol/L vs. 82.9 ± 27.3 nmol/L, *P* = 0.014).

Mean baseline serum zinc level was lower in the Opt 2.0 group compared with the Opt 1.0 group (11.2 ±2.3 μmol/L vs. 12.2 ±1.6 μmol/L, *P *= 0.003), and the prevalence of zinc deficiencies at baseline was also higher in this group (respectively 17.1% vs. 1.6%, *P *= 0.003). In spite of the lower level at baseline, at T6 mean serum zinc level was higher in the Opt 2.0 group than in the Opt 1.0 group (12.9 ±2.2 μmol/L vs. 11.7 ±1.6 μmol/L, *P *= 0.003). ). Over time, zinc levels increased in the Opt 2.0 group but decreased in the Opt 1.0 group (+1.3 ±3.9 μmol/L vs. -0.4 ±2.2 μmol/L, *P* < 0.001). The prevalence of zinc deficiencies at T6 and T12 did not differ between the groups (*P* > 0.05 for both).

No significant differences were observed for vitamin B1. Results of the PP analysis were similar to the results of the ITT analysis (Supplementary Table 3).

### Hypervitaminosis

The prevalence of hypervitaminosis was similar between the two groups. Overall, serum levels above the reference values were observed for ferritin (11.2%), folic acid (20.8%), vitamin B1 (19.2%), and vitamin B6 (39.4%) throughout the study period. Serum levels above the reference values for all other micronutrients were rare (< 3%).

For vitamin B1, about one third of the patients (35%) already presented with excess serum levels at baseline, whereas hypervitaminosis for vitamin B6 developed de novo after surgery in more than three quarters of patients (79%) with excess vitamin B6 levels. Extremely high serum vitamin B6 levels (> 200 nmol/L) were only found in one patient.

## Discussion

The present study evaluated the short-term effectiveness of the optimized WLS Optimum 2.0 supplement on preventing micronutrient deficiencies after SG in comparison to its previous version, WLS Optimum 1.0. WLS Optimum 2.0 contained higher levels of elementary iron, folic acid, vitamin B12, vitamin B1, copper, and zinc and a lower level of vitamin A than WLS Optimum 1.0.

We found higher serum levels of vitamin B12, vitamin B6, and zinc and a lower prevalence of deficiencies for vitamin B12 and phosphate in the Opt 2.0 group. MCV and serum folic acid levels were higher in the Opt 1.0 group. Over the 12-month study period, mean increase in serum levels of phosphate, vitamin B6, and zinc was higher in the Opt 2.0 group and MCV and serum vitamin D levels increased more in the Opt 1.0 group. According to the PP analysis, we additionally found that the mean increase in PTH and serum calcium levels were higher in the Opt 2.0 group whereas the mean increase in serum albumin levels was lower in this group.

The level of elementary iron was increased from 21 mg to 28 mg. Although we found that serum ferritin levels equally increased in both groups, 11% of the patients in the Opt 2.0 group was iron deficient during the study period compared to 3% in the Opt 1.0 group. In the PP analysis, only 3% of the patients in the Opt 2.0 group were iron deficient. This indicates that most of the iron deficiencies occurred in non-compliant patients. In the Opt 1.0 group, the prevalence of iron deficiency did not change in the PP analysis. Our findings therefore suggest that a level of 28 mg of elementary iron is sufficient to prevent deficiencies at 12 months post-surgery. According to the nutritional guidelines of the American Society for Metabolic and Bariatric Surgery (ASMBS), patients who have undergone RYGB or SG should take at least 45–60 mg of elemental iron daily [15]. Based on our findings, this recommendation should be revised for SG patients as high doses of elementary iron may increase the risk of adverse gastrointestinal side effects [16, 17].

The level of folic acid was increased from 300 to 500 μg. Mean increase in serum folic acid level and the prevalence of folic acid deficiencies were similar between the groups according to the ITT analysis. However, mean increase in serum folic acid level was twice as high in the Opt 2.0 group compared with the Opt 1.0 group in the PP analysis (+11.2 nmol/L vs. +6.8 nmol/L, *P *= 0.11). Our results indicate that 500 μg of folic acid is sufficient, which is in line with the ASMBS recommendation of 400–800 μg per day [15].

The 10-fold increase in vitamin B12 (10–100 μg) was clearly reflected in significantly higher serum levels and less vitamin B12 deficiencies in the Opt 2.0 group compared with the Opt 1.0 group. The ASMBS recommendation for vitamin B12 is 350–500 μg oral supplementation per day for all bariatric patients, irrespective of the type of weight loss surgery [15]. Based on our results, it would better to distinguish between the different types of surgery as our data indicate that a lower vitamin B12 level of 100 μg is sufficient to maintain adequate serum vitamin B12 levels in SG patients. Our findings for vitamin B12 could also explain why we found a higher mean increase in MCV in the Opt 1.0 group than in the Opt 2.0 group (+ 3.2 ± 4.2 fL vs. + 0.9 ± 3.4 fL, *P* = 0.002). Mean corpuscular volume (MCV) is a laboratory value that measures the average size and volume of a red blood cell [18]. MCV below the lower limit of 80 fL can indicate iron deficiency anemia, while MCV above the upper limit of 100 fL is associated with vitamin B12 deficiency [18]. In the present study, the increase in MCV in the Opt 1.0 group was indeed accompanied by a decrease in serum vitamin B12 level in this group. Moreover, the change in MCV was significantly correlated with the change in serum vitamin B12 level (r = −0.32, *P* < 0.001).

The level of vitamin B1 was increased from 2.00 to 2.75 mg in Optimum 2.0. Although mean serum vitamin B1 levels decreased in both groups, deficiencies were rare. In fact, hypervitaminosis for vitamin B1 was more prevalent than vitamin B1 deficiency in the present study (19% vs. 4%, respectively). Despite the fact that complications of high doses of vitamin B1 are rare as the body can excrete excess amounts of thiamin in the urine [19], the ASMBS recommendation of at least 12 mg vitamin B1 per day seems highly overestimated and should be revised [15]. Since mean serum vitamin B1 decreased less in the Opt 2.0 group, the dose of 2.75 mg is preferred over the dose of 2.00 mg.

The level of zinc was nearly doubled from 15 to 28 mg. This resulted in a larger increase in mean serum zinc level in the Opt 2.0 group than in the Opt 1.0 group. The ASMBS recommendation for zinc is 8–11 mg per day for SG patients [15]. This seems highly underestimated as we found that mean serum zinc level decreased in patients using Optimum 1.0, which contained 15 mg of zinc. Despite the high level of zinc in Optimum 2.0, only one patient in the Opt 2.0 group slightly exceeded the upper limit of the reference range (level of 19.6 μmol/L). Acute adverse effects of excess zinc include epigastric pain, nausea, vomiting, loss of appetite, abdominal cramps, diarrhea, and headaches [20]. On the long term, excessive absorption of zinc can suppress copper and iron absorption [20]. The Tolerable Upper Intake Level for zinc is 40 mg per day [20]. Basfi-Fer et al. found that dietary intake of zinc varied between 6.3–8.4 mg per day in the first 2 years post-sleeve [21]. It would hence appear safe to recommend 28 mg zinc for these patients without any significant risk of zinc toxicity. The level of copper in WLS Optimum 2.0 was also increased from 1.0 to 1.9 mg to maintain the recommended ratio of 15 mg of zinc to 1 mg of copper [22].

Whereas no vitamin B6 deficiencies were observed, hypervitaminosis for vitamin B6 was highly prevalent in both groups (39%). This is in line with previous research reporting a low prevalence of vitamin B6 deficiency (0–0.5%) but excess vitamin B6 levels in up to 50% of the patients [7, 8, 14, 23]. Excessive serum levels of vitamin B6 can cause neuropathic symptoms [24], but the toxicity of vitamin B6 may depend on which form of vitamin B6 is used in a supplement. Vrolijk et al. found that the neuropathy observed after taking a relatively high dose of vitamin B6 supplements is due to pyridoxine [25]. They suggested to replace pyridoxine by pyridoxal or pyridoxal-phosphate in vitamin B6 supplements, since they are much less toxic [25]. Therefore, the vitamin B6 vitaminer was changed from pyridoxine in Optimum 1.0 to pyridoxal-phosphate in Optimum 2.0. This could also explain why we found a higher increase in serum vitamin B6 level in the Opt 2.0 group than in the Opt 1.0 group. Unlike pyridoxine, pyridoxal-phosphate is the active coenzyme form of vitamin B6 which can be directly utilized by the body without conversion [25]. In order to decrease the risk of adverse effects from excess vitamin B6 serum levels, the level of vitamin B6 in WLS Optimum should be decreased to 1.5 mg pyridoxal-phosphate.

The prevalence of phosphate deficiencies was significantly higher in the Opt 1.0 group than in the Opt 2.0 group at T6 (20.4% vs. 4.8%, *P*=0.028). This significant difference might be explained by a change in the reference value for phosphate halfway during the VITAAL I RCT. The reference value for phosphate was changed from 0.87–1.45 mmol/L to 0.80–1.40 mmol/L because of a new assay. However, all patients in the VITAAL I RCT were analyzed by using the old reference value. When using the correct reference value for each individual patient in the VITAAL I RCT, prevalence of phosphate deficiency was 21% at T0, 8.2% at T6, and 10.9% at T12 in the Opt 1.0 group. These rates are no longer significantly different from those in the Opt 2.0 group.

Overall, we found that observed differences in serum levels and prevalence of deficiencies between the two groups were most pronounced in the PP analysis. However, most of these results did not reach statistical significance, which might have been because of a reduced power due to the small sample of compliant patients. Unfortunately, lifelong compliance with a daily multivitamin supplement is hard to achieve in this patient population [26]. In the present study, compliance rates decreased to 55% at 12 months in both groups. The main reported reason for discontinuation of the assigned MVS was nausea. Most patients switched to a regular over-the-counter MVS, but others did not tolerate any MVS and therefore stopped using multivitamin supplementation. More research is needed to explore the underlying factors in order to increase patient compliance.

Although all study participants received the supplements free-of-charge, the costs of treatment with specialized MVS have also been considered a major barrier to adequate lifelong adherence [26]. Compared with the price of other commercially available bariatric multivitamin formulations, WLS Optimum is in the mid-range with a price of €0.29 per capsule. Whereas the use of such supplements initially seems more expensive, Homan et al. showed that the use of a specialized multivitamin resulted in less overall costs compared with using sMVS [27].

Because of the large variety in composition of bariatric multivitamin formulations, this study can contribute towards the achievement of the most optimal form and content of bariatric MVS. However, it is important to note that although our data can give an indication about the doses needed to prevent deficiencies and hypervitaminosis, longer-term follow-up studies are necessary to confirm our findings. In that respect, it would also be very useful if data of other MVS formulations become available.

One of the strengths of the present study was the performance of both an ITT analysis and PP analysis based on self-reported compliance. In this way, we could establish the efficacy in a real-life setting as well as in an ideal setting in which all patients adhere to the supplement protocol. Furthermore, we were able to minimize the risk of bias related to a difference in supplements as they were produced by the same manufacturer.

Limitations include the absence of information on nutritional intake and the lack of a (randomized) control group in the current study. The VITAAL II study was a single-arm open label study and we compared this group to the intervention group of the VITAAL I RCT. Although these studies were performed in different time periods, the operative technique, surgeons, researchers, and hospital were the same in both studies. Moreover, both groups were similar with respect to age, gender, preoperative body weight and BMI, and comorbidities

Another limitation is that according to clinical practice, only preoperative deficiencies for vitamin B12 and vitamin D were treated. Not correcting for all preoperative deficiencies could have affected our findings regarding the efficacy of both multivitamin supplements. We therefore corrected for baseline serum levels in the statistical analyses. Moreover, we excluded serum level data of patients who used additional supplementation to prevent biased estimates. However, information on the intake of additional supplementation was subjective (via self-report and medical files) and probably, despite an extensive check, not complete which could also have influenced the results.

## Conclusion

The present study showed that the use of a specialized multivitamin supplement for SG patients (WLS Optimum 2.0) is effective at preventing deficiencies for most vitamins and minerals, specifically in compliant patient. The level of vitamin B6 should be lowered from 2 to 1.5 mg pyridoxal-phosphate to decrease the risk of vitamin B6 toxicity. A strict follow-up regime remains necessary to be able to diagnose nutritional deficiencies as well as hypervitaminosis in an early stage and to improve patients’ compliance with a daily multivitamin supplement. More research is needed to identify which factors affect compliance and how this can be improved.

## Supplementary Information

ESM 1 (DOCX 21 kb)

## References

[1] Ramos A, Kow L, Brown W, et al. Fifth IFSO Global Registry Report. 2019.

[2] Coupaye M, Sami O, Calabrese D, et al. Prevalence and determinants of nutritional deficiencies at mid-term after sleeve gastrectomy. Obes Surg. 2020. 10.1007/s11695-020-04425-3 32016653

[3] Ruiz-Tovar J, Llavero C, Zubiaga L, Boix E (2016). group O. Maintenance of multivitamin supplements after sleeve gastrectomy. Obes Surg.

[4] Kehagias I, Zygomalas A, Karavias D, Karamanakos S (2016). Sleeve gastrectomy: have we finally found the holy grail of bariatric surgery? A review of the literature. Eur Rev Med Pharmacol Sci.

[5] Lupoli R, Lembo E, Saldalamacchia G, Avola CK, Angrisani L, Capaldo B (2017). Bariatric surgery and long-term nutritional issues. World J Diabetes.

[6] Sawaya RA, Jaffe J, Friedenberg L, Friedenberg FK (2012). Vitamin, mineral, and drug absorption following bariatric surgery. Curr Drug Metab.

[7] Aarts EO, Janssen IM, Berends FJ (2011). The gastric sleeve: losing weight as fast as micronutrients?. Obes Surg.

[8] Al-Mutawa A, Al-Sabah S, Anderson AK, Al-Mutawa M (2017). Evaluation of nutritional status post laparoscopic sleeve gastrectomy—5-year outcomes. Obes Surg.

[9] Ben-Porat T, Elazary R, Goldenshluger A, Sherf Dagan S, Mintz Y, Weiss R (2017). Nutritional deficiencies four years after laparoscopic sleeve gastrectomy-are supplements required for a lifetime?. Surg Obes Relat Dis.

[10] Caron M, Hould FS, Lescelleur O, Marceau S, Lebel S, Julien F, Simard S, Biertho L (2017). Long-term nutritional impact of sleeve gastrectomy. Surg Obes Relat Dis.

[11] Gillon S, Jeanes YM, Andersen JR, Vage V (2017). Micronutrient status in morbidly obese patients prior to laparoscopic sleeve gastrectomy and micronutrient changes 5 years post-surgery. Obes Surg.

[12] Mechanick JI, Apovian C, Brethauer S, et al. Clinical practice guidelines for the perioperative nutrition, metabolic, and nonsurgical support of patients undergoing bariatric procedures - 2019 update: cosponsored by American Association of Clinical Endocrinologists/American College of Endocrinology, The Obesity Society, American Society for Metabolic & Bariatric Surgery, Obesity Medicine Association, and American Society of Anesthesiologists. Surg Obes Relat Dis. 2019. 10.1016/j.soard.2019.10.025 31917200

[13] Heusschen L, Schijns W, Ploeger N, et al. The True Story on Deficiencies After Sleeve Gastrectomy: Results of a Double-Blind RCT. Obes Surg. 2019. 10.1007/s11695-019-04252-1 31776782

[14] van Rutte PW, Aarts EO, Smulders JF, Nienhuijs SW (2014). Nutrient deficiencies before and after sleeve gastrectomy. Obes Surg.

[15] Parrott J, Frank L, Rabena R, Craggs-Dino L, Isom KA, Greiman L (2017). American Society for Metabolic and Bariatric Surgery Integrated Health Nutritional Guidelines for the Surgical Weight Loss Patient 2016 Update: Micronutrients. Surg Obes Relat Dis.

[16] Cancelo-Hidalgo MJ, Castelo-Branco C, Palacios S, Haya-Palazuelos J, Ciria-Recasens M, Manasanch J, Pérez-Edo L (2013). Tolerability of different oral iron supplements: a systematic review. Curr Med Res Opin.

[17] Tolkien Z, Stecher L, Mander AP, Pereira DI, Powell JJ (2015). Ferrous sulfate supplementation causes significant gastrointestinal side-effects in adults: a systematic review and meta-analysis. PLoS One.

[18] Maner BS, Moosavi L (2020). Mean Corpuscular Volume (MCV).

[19] Kerns JC, Gutierrez JL (2017). Thiamin. Adv Nutr.

[20] Trumbo P, Yates AA, Schlicker S, Poos M (2001). Dietary reference intakes: vitamin A, vitamin K, arsenic, boron, chromium, copper, iodine, iron, manganese, molybdenum, nickel, silicon, vanadium, and zinc. J Am Diet Assoc.

[21] Basfi-Fer K, Rojas P, Carrasco F, Valencia A, Inostroza J, Codoceo J (2012). Evolution of the intake and nutritional status of zinc, iron and copper in women undergoing bariatric surgery until the second year after surgery. Nutr Hosp.

[22] Osredkar J, Sustar N (2011). Copper and zinc, biological role and significance of copper/zinc imbalance. J Clin Toxicolo.

[23] Smelt HJM, van Loon S, Pouwels S, Boer AK, Smulders JF, Aarts EO (2020). Do Specialized Bariatric Multivitamins Lower Deficiencies After Sleeve Gastrectomy?. Obes Surg.

[24] Alsabah A, Al Sabah S, Al-Sabah S, Al-Serri A, Al Haddad E, Renno WM (2017). Investigating Factors Involved in Post Laparoscopic Sleeve Gastrectomy (LSG) Neuropathy. Obes Surg.

[25] Vrolijk MF, Opperhuizen A, Jansen E, Hageman GJ, Bast A, Haenen G (2017). The vitamin B6 paradox: Supplementation with high concentrations of pyridoxine leads to decreased vitamin B6 function. Toxicol in Vitro.

[26] Smelt HJM, Pouwels S, Smulders JF, et al. Patient adherence to multivitamin supplementation after bariatric surgery: a narrative review. J Nutr Sci. 2020;9 10.1017/jns.2020.41 PMC7550964 33101663

[27] Homan J, Schijns W, Janssen IMC, Berends FJ, Aarts EO (2019). Adequate Multivitamin Supplementation after Roux-En-Y Gastric Bypass Results in a Decrease of National Health Care Costs: a Cost-Effectiveness Analysis. Obes Surg.

