# Supplementary material for: Optimizing Multivitamin Supplementation for Sleeve Gastrectomy Patients
Source: Obes Surg. 2021 Feb 23;31(6):2520–8. doi: 10.1007/s11695-021-05282-4 (PMC8113195; doi:10.1007/s11695-021-05282-4)
Supplement: Supplementary file 1 — (DOCX 21 kb) [file 11695_2021_5282_MOESM1_ESM.docx]

**Supplementary Table 1.** Per-protocol results of mean serum levels and prevalence of deficiencies of Hemoglobin, MCV, Ferritin, Folic acid and Vitamin B12.

| **Serum variables**  **(reference values)** | **Type MVS** | **Serum levels** | | | **Deficiencies** | |
| --- | --- | --- | --- | --- | --- | --- |
|  |  | **T6** | **T12** | **Δ (T12-T0)** | **T6** | **T12** |
| **Hemoglobin**  (M: 8.4 – 10.8 mmol/L  F: 7.4 – 9.9 mmol/L) | **Optimum 1.0**  (n=44/38) | 8.7 ± 0.7 | 8.5 ± 0.6 | -0.3 ± 0.7 | 0 (0.0) | 1 (2.6) |
|  | **Optimum 2.0**  (n=46/35) | 8.7 ± 0.7 | 8.7 ± 0.8 | -0.3 ± 0.6 | 3 (6.5) | 2 (5.7) |
| **MCV**  (80-100 fL) | **Optimum 1.0**  (n=44/38) | 90.2 ± 3.1 | 91.7 ± 3.9 | +2.1 ± 2.9 | 0 (0.0) | 0 (0.0) |
|  | **Optimum 2.0**  (n=44/35) | 89.6 ± 3.5 | 89.6 ± 3.7^*^ | +0.8 ± 3.1 | 0 (0.0) | 0 (0.0) |
| **Ferritin**  (20-300 ng/mL) | **Optimum 1.0**  (n=37/37) | 148.3 ± 116.4 | 150.0 ± 116.5 | +2.6 ± 60.3 | 0 (0.0) | 1 (2.7) |
|  | **Optimum 2.0**  (n=45/34) | 151.5 ± 105.3 | 151.0 ± 112.6 | +2.4 ± 55.6 | 1 (2.2) | 1 (2.9) |
| **Folic acid**  (6-28 nmol/L)^1^ | **Optimum 1.0**  (n=44/38) | 24.1 ± 8.7 | 24.4 ± 10.3 | +6.8 ± 9.7 | 0 (0.0) | 1 (2.6) |
|  | **Optimum 2.0**  (n=45/34) | 21.5 ± 10.1 | 26.6 ± 14.1 | +11.2 ± 13.8 | 1 (2.2) | 1 (2.9) |
| **Vitamin B12**  (200-570 pmol/L) | **Optimum 1.0**  (n=36/33) | 278.9 ± 90.0 | 277.5 ± 77.8 | -25.3 ± 83.2 | 8 (22.2) | 7 (21.2) |
|  | **Optimum 2.0**  (n=43/29) | 312.6 ± 105.1 | 322.2 ± 98.4^*^ | +19.3 ± 100.6 | 6 (14.0) | 1 (3.4) |

Data are presented as mean ± standard deviation and frequencies (percentages). *MVS,* multivitamin supplement; *MCV* mean corpuscular volume.

^1^Reference range for the used assay in the VITAAL II study (Optimum 2.0) was 6-28 ng/mL

^*^*P*<0.05 for Optimum 1.0 vs. Optimum 2.0.

**Supplementary Table 2.** Per-protocol results of mean serum levels and prevalence of deficiencies of Vitamin D, PTH, Calcium, Magnesium, Phosphate and Albumin.

| **Serum variables**  **(reference values)** | **Type MVS** | **Serum levels** | | | | **Deficiencies** | |
| --- | --- | --- | --- | --- | --- | --- | --- |
|  |  | **T6** | **T12** | | **Δ (T12-T0)** | **T6** | **T12** |
| **Vitamin D**  (>50 nmol/L) | **Optimum 1.0**  (n=44/37) | 87.4 ± 25.2 | 88.0 ± 28.4 | | +48.2 ± 28.2 | 1 (2.3) | 2 (5.4) |
|  | **Optimum 2.0**  (n=44/35) | 93.9 ± 24.8 | 88.5 ± 20.6 | | +30.8 ± 23.0^*^ | 0 (0.0) | 1 (2.9) |
| **PTH**  (1.3 – 6.8 pmol/L) | **Optimum 1.0**  (n=44/37) | 3.1 ± 1.4 | 3.2 ± 1.7 | | -0.3 ± 1.9 | 0 (0.0)^1^ | 2 (5.4)^1^ |
|  | **Optimum 2.0**  (n=45/34) | 3.1 ± 1.8 | 3.9 ± 2.2 | | +0.5 ± 1.4^*^ | 1 (2.2)^1^ | 3 (8.8)^1^ |
| **Calcium**^2^  (2.10-2.55 mmol/L)^3^ | **Optimum 1.0**  (n=43/37) | 2.39 ± 0.08 | 2.41 ± 0.09 | | +0.05 ± 0.11 | 0 (0.0) | 0 (0.0) |
|  | **Optimum 2.0**  (n=46/34) | 2.42 ± 0.08 | 2.39 ± 0.09 | | +0.06 ± 0.09^*^ | 0 (0.0) | 1 (2.9) |
| **Magnesium**  (0.70-1.10 mmol/L) | **Optimum 1.0**  (n=29/27) | 0.82 ± 0.04 | 0.82 ± 0.05 | | +0.02 ± 0.07 | 0 (0.0) | 0 (0.0) |
|  | **Optimum 2.0**  (n=27/14) | 0.82 ± 0.06 | 0.81 ± 0.06 | | +0.03 ± 0.05 | 1 (3.7) | 0 (0.0) |
| **Phosphate**  (0.87-1.45 mmol/L)^4^ | **Optimum 1.0**  (n=36/33) | 1.01 ± 0.16 | 1.03 ± 0.22 | | +0.08 ± 0.20 | 7 (19.4) | 7 (21.2) |
|  | **Optimum 2.0**  (n=30/19) | 1.04 ± 0.14 | 1.08 ± 0.21 | | +0.22 ± 0.19^**^ | 1 (3.3) | 1 (5.3) |
| **Albumin**  **(**35-50 g/L) | **Optimum 1.0**  (n=43/37) | 38.8 ± 3.2 | 38.6 ± 2.6 | | +1.3 ± 3.0 | 4 (9.3) | 2 (5.4) |
|  | **Optimum 2.0**  (n=46/34) | 38.2 ± 3.0 | | 38.5 ± 3.1 | -0.03 ± 2.6^*^ | 4 (8.7) | 4 (11.8) |

Data are presented as mean ± standard deviation and frequencies (percentages). *MVS,* multivitamin supplement; *PTH,* parathyroid hormone

^1^ Elevated PTH levels.

^2^ Corrected for albumin levels (total calcium – (0.025 x albumin) + 1).

^3^ Reference range for the used assay in the VITAAL II study (Optimum 2.0) was 2.20-2.65 mmol/L

^4^ Reference range for the used assay in the VITAAL II study (Optimum 2.0) was 0.80-1.40 mmol/L

^*^*P*<0.05, ^**^*P*<0.01 for Optimum 1.0 vs. Optimum 2.0.

**Supplementary Table 3.** Per-protocol results of mean serum levels and prevalence of deficiencies of Vitamin B1 and B6, and Zinc

| **Serum variables**  **(reference values)** | **Type MVS** | **Serum levels** | | | **Deficiencies** | |
| --- | --- | --- | --- | --- | --- | --- |
|  |  | **T6** | **T12** | **Δ (T12-T0)** | **T6** | **T12** |
| **Vitamin B1**  (95-175 nmol/L) | **Optimum 1.0**  (n=36/33) | 150.2 ± 27.6 | 146.9 ± 33.2 | -21.0 ± 46.6 | 0 (0.0) | 2 (6.1) |
|  | **Optimum 2.0**  (n=28/16) | 149.9 ± 26.6 | 156.1 ± 29.8 | -8.6 ± 41.8 | 0 (0.0) | 0 (0.0) |
| **Vitamin B6**  (25-100 nmol/L) | **Optimum 1.0**  (n=36/33) | 93.8 ± 39.0 | 84.9 ± 29.4 | +7.1 ± 26.6 | 0 (0.0) | 0 (0.0) |
|  | **Optimum 2.0**  (n=28/15) | 90.4 ± 25.9 | 105.7 ± 27.8^*^ | +33.7 ± 28.2^*^ | 0 (0.0) | 0 (0.0) |
| **Zinc**  (9.2-18.4 µmol/L) | **Optimum 1.0**  (n=35/33) | 11.9 ± 1.7 | 11.8 ± 2.0 | -0.2 ± 2.2 | 2 (5.7) | 0 (0.0) |
|  | **Optimum 2.0**  (n=28/15) | 13.5 ± 2.1^***^ | 12.9 ± 2.2 | +2.0 ± 4.1^***^ | 0 (0.0) | 1 (6.7) |

Data are presented as mean ± standard deviation and frequencies (percentages). *MVS,* multivitamin supplement.

^*^*P*<0.05, ^***^*P*<0.001 for Optimum 1.0 vs. Optimum 2.0.
